# Transcriptomic and Metabolomic Responses of Rice Plants to *Cnaphalocrocis medinalis* Caterpillar Infestation

**DOI:** 10.3390/insects11100705

**Published:** 2020-10-15

**Authors:** Yuqi Wang, Qingsong Liu, Lixiao Du, Eric M. Hallerman, Yunhe Li

**Affiliations:** 1 State Key Laboratory for Biology of Plant Diseases and Insect Pests, Institute of Plant Protection, Chinese Academy of Agricultural Sciences, Beijing 100193, China; wangyuqi2012nankai@126.com (Y.W.); liuqingsong@xynu.edu.cn (Q.L.); dulixiao_1988@163.com (L.D.); 2 College of Life Sciences, Xinyang Normal University, Xinyang 464000, China; 3 Department of Fish and Wildlife Conservation, Virginia Polytechnic Institute and State University, Blacksburg, VA 24061, USA; ehallerm@vt.edu

**Keywords:** transcriptome, metabolome, jasmonate pathway, transcription factor, plant-insect interaction

## Abstract

**Simple Summary:**

The transcriptomic and metabolomic differences in rice leaves after infestation by the rice leaf folder *Cnaphalocrocis medinalis* were investigated for better understanding of the mechanisms of rice defenses against this species. The results suggest that *C. medinalis* infestation can induce rapid and precise defense responses involved in many primary and secondary metabolic processes in rice leaves, and the jasmonic acid (JA)-dependent signaling pathway plays vital roles in the response of rice plants to this pest species. These results provide comprehensive insights into the defense system of rice to the rice leaf folder and may facilitate the development of insect-resistant rice varieties by identifying molecular targets for selection.

**Abstract:**

Interactions between plants and insect herbivores are important determinants of plant productivity in cultivated and natural agricultural fields. The rice leaf folder (*Cnaphalocrocis medinalis*) causes tremendous damage to rice production in Asian countries. However, little information is available about how rice plants defend themselves against this destructive pest at molecular and biochemical levels. Here, we observed the transcriptomic and metabolomic differences in rice leaves after 0, 1, 6, 12, and 24 h of being fed by *C. medinalis* using RNA sequencing and metabolome profiling. Transcriptional analyses showed that gene expression responds rapidly to leaf folder infestation, with the most significant transcriptional changes occurring within 6 h after the initiation of feeding. Metabolite abundance changed more slowly than gene expression. Gene Ontology and Kyoto Encyclopedia of Genes and Genomes enrichment analyses indicated that the rice transcriptional response to infestation involved genes encoding protein kinases, transcription factors, biosynthesis of secondary metabolites, photosynthesis, and phytohormone signaling. Moreover, the jasmonic acid-dependent signaling pathway triggered by leaf folder herbivory played a vital role in rice defense against this pest. Taken together, our results provide comprehensive insights into the defense system of rice to this species and may inform the development of insect-resistant rice varieties.

## 1. Introduction

The intensive interactions between insect herbivores and their plant hosts during infestation can result in plant physiological disorders and even death, posing major threats to crop yield and food security worldwide. During the coevolutionary arms race between plants and insects, plant hosts have evolved multiple mechanisms of defense against insects, including direct and indirect responses. The direct defense relies on resistance factors expressed by the plant itself that directly and negatively affect the growth, development, and fecundity of insect herbivores [1]. While the indirect defense involves the release of herbivore-induced plant volatiles (HIPVs) and nectar rewards that attract natural enemies of the herbivorous insects [1,2,3], the combination of direct and indirect defense provides durable resistance to a broad spectrum of herbivores in natural ecosystems [2].

Plant defense against insect attack is a dynamic, multidimensional process, starting with the recognition of herbivores, followed by the induction of signal transduction pathways and the reprogramming of the transcriptome, proteome, and metabolome, and finally with the production of defense compounds, proteins, and secondary metabolites, ultimately leading to changes in plant phenotype [4,5]. The elicitation of defense responses depends upon the rapid and accurate recognition of herbivore-associated molecular patterns. Early signaling events triggered by herbivory are the initial response in plants and are responsible for triggering downstream signaling transduction pathways [1,2,6]. These signaling events mainly include depolarization of membrane potential, calcium flux, production of reactive oxygen species, and mitogen-activated protein kinase (MAPK) activity [2,6]. In the dynamic defense process, the plant hormonal signaling network connects perception and early signaling events to broad transcriptional reorganization and defense induction [6]. Plant hormones, including jasmonic acid (JA), salicylic acid (SA), ethylene (ET), cytokinins, abscisic acid (ABA), gibberellins, and auxin, play important roles in herbivore-induced defense signaling. Among these phytohormones, the JA and SA pathways are well studied [2,6,7]. The JA pathway is usually activated by chewing insects and cell-content feeders, while the SA pathway is primarily induced by piercing-sucking herbivores. In dicotyledonous plants, the JA pathway is well established as the core and conserved pathway that regulates plant resistance to a broad spectrum of insects [1,2,6]. However, the role of the JA pathway in monocots, such as rice, remains largely unknown.

Rice (*Oryza sativa*) is an important crop plant worldwide and feeds more than half of the world’s population [8]. However, rice production is greatly restricted by herbivorous insects. It has been estimated that more than 200 species of insect pests bring a 20–40% yield loss annually [9]. Among the numerous insect pests of rice, the leaf folder *Cnaphalocrocis medinalis* Guenée (Family Crambidae) is the most important foliage-feeding lepidopteran species [10,11,12]. *C. medinalis* is a migratory pest in all the major rice-growing regions in Asia, including China, India, Japan, Korea, Malaysia, Sri Lanka, and Vietnam [12,13]. The larvae of *C. medinalis* cause damage by folding and defoliating the rice leaves. In recent years, frequent outbreaks of this pest have been reported throughout rice-growing regions of China and have greatly threatened rice production [10]. Currently, the application of broad-spectrum insecticides is the main strategy for the control of *C. medinalis*. However, such pest management practices can reduce farm profitability, affect human and environmental health, and lead to increased insecticide resistance and pest resurgence [13].

A full understanding of the genetic and molecular bases of rice defense against *C. medinalis* is important for informing the development of efficient strategies to control this pest. Previously, it has been demonstrated that application of methyl jasmonate (MeJA) and methyl salicylate (MeSA) as topical sprays onto rice plants significantly improve rice defense against *C. medinalis* [14,15]. Recently, it was found that rice leaf rolling by *C. medinalis* larvae, but not by artificial leaf rolling, attracts the parasitoid *Itoplectis naranyae* as an indirect defense [16]. It is well known that plant response to herbivore damage is a dynamic process and that this process is associated with large-scale change at the mRNA, protein, and metabolite levels [5,17]. However, few studies have been conducted to clarify the defense mechanisms of rice against *C. medinalis* feeding at the molecular level, and dynamic defense responses following the onset of infestation by this herbivore are still unclear.

In the current study, we investigated the dynamic rice responses to *C. medinalis* feeding by integrating results from high-throughput RNA-sequencing (RNA-seq) with phytohormone and metabolome profiling experiments to advance understanding of rice defense mechanisms to *C. medinalis* infestation. Our findings may facilitate the development of new approaches for crop protection and improvement.

## 2. Materials and Methods

### 2.1. Plants and Insets

Rice seeds (strain Minghui 63) were provided by Professor Hongxia Hua (Huazhong Agricultural University, Wuhan, China). The rice seeds were incubated in water for 2 days and were sown in a greenhouse under a controlled photoperiod of 16:8 h (light:dark) at 27 ± 3 °C with 75 ± 10% relative humidity (RH). After 2 weeks, the seedlings were transplanted into individual plastic pots (diameter 10 cm, height 8 cm), containing a 3:1 mixture of peat and vermiculite (Meihekou Factory, Meihekou, China). The potted plants were placed in a cement pool at the Langfang Experimental Station of the Institute of Plant Protection, Chinese Academy of Agricultural Sciences (CAAS) in Hebei Province. Sakefu nitrogenous fertilizer (N [20%], P_2_O_5_ [20%], K_2_O [20%]; Sino-Arab Chemical Fertilizer Co., Ltd., Qinhuangdao, China) was applied weekly. Plants were grown for 5 weeks and were then used for the experiments.

Specimens of *C. medinalis* used in the experiments were obtained from a laboratory colony. The colony was maintained in the laboratory since May 2013 with annual introduction of field-collected individuals. The larvae were reared with 15-day-old Zhengdan958 maize seedlings, and 3rd-instar larvae were used in the experiments. The colony was maintained in a climate-controlled chamber at 27 ± 1 °C, 75 ± 10% RH and a photoperiod of 16:8 h (light:dark).

### 2.2. Sample Preparation

**Figure 1:**
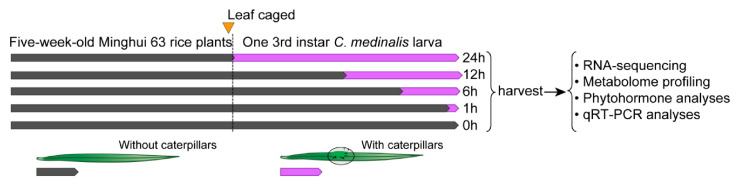
Design of the rice leaf folder feeding experiments. The 2nd leaf from the top of one 5-week-old Minghui 63 rice plant was enclosed in a Parafilm bag. At staggered intervals, one rice leaf folder *C. medinalis* caterpillar was added to each bag. Leaf tissue was harvested after 0–24 h of leaf folder feeding. Harvested samples were immediately frozen in liquid nitrogen and stored at −80 °C for further processing.

A soft brush was used to place one 3rd-instar *C. medinalis* on the second leaf from the top. The larvae were starved for 2 h before experiments and confined within a small Parafilm bag [18]. For the time-course bioassays, the larvae were transferred to rice leaves for the final 1, 6, 12, or 24 h of the experiment (Figure 1). Control rice plants were placed in the same type of Parafilm bags as in the herbivory treatment but without placement of insects. All leaf materials were harvested at the same time. The samples were immediately frozen in liquid nitrogen and stored at −80 °C for further use. Five leaf tissues collected from five independent plants within the same treatments were merged as one sample (i.e., biological replicate). Four samples (i.e., replicates) were collected at each time point for transcriptome analyses, and three samples were collected at each time point for metabolome analyses.

### 2.3. RNA Isolation, Library Construction, Sequencing, and Validation

Total RNA from rice leaf samples was isolated using TRIzol Reagent (Life Technologies, Grand Island, NY, USA), following the manufacturer’s instructions. RNA degradation and contamination were assessed using 2% agarose gels. The quality and quantity of the RNA were checked using a NanoPhotometer^®^ spectrophotometer (Implen, Westlake Village, CA, USA) and a Qubit^®^ 2.0 Fluorometer (Life Technologies, Carlsbad, CA, USA), respectively. RNA integrity was assessed using an Agilent RNA Nano 6000 Assay Kit with an Agilent Bioanalyzer 2100 system (Agilent Technologies, Santa Clara, CA, USA). RNA samples with a RNA Integrity Number (RIN) ≥ 7.1 were used in the subsequent experiments. Libraries were prepared using the NEBNext^®^ Ultra^TM^ RNA Library Prep Kit for Illumina^®^ (New England Biolabs, Ipswich, MA USA), according to the manufacturer’s instructions, and were sequenced on an Illumina sequencing platform (Illumina Hiseq 4000; Illumina, San Diego, CA, USA), and 125–150 bp paired-end reads were generated.

To validate the results of RNA-seq, eight genes involved in the JA pathway were selected for quantitative real-time PCR (qRT-PCR) analyses. Total RNA was extracted using TRIzol reagent (Invitrogen, Carlsbad, CA, USA), and cDNA was synthesized from total RNA using TransScript^®^ One-Step gDNA Removal and cDNA Synthesis SuperMix (TransGen Biotech, Beijing, China). Primer 5 software (Premier Biosoft International, Palo Alto CA, USA) was used to design gene-specific primers (Table S1). qRT-PCR was performed on a Bio-RadCFX96 Touch Real-time PCR Detection System instrument (Bio-Rad, Hercules, CA, USA) using *TransStart*^®^ Top Green qPCR SuperMix (TransGen Biotech, Beijing, China), according to the manufacturer’s protocol. The PCR amplification program was the following: 30 s at 94 °C, followed by 40 cycles of 5 s at 94 °C and 30 s at 60 °C. The relative expression values were obtained using the 2^−ΔΔCt^ method with *ubiquitin 5* as a reference. The qRT-PCR reactions were repeated using three biological and two technical replications.

### 2.4. RNA-Sequencing Data Analysis

To obtain high-quality reads, raw reads containing adapter, poly-N, or low-quality bases (Phred quality score Q < 20) were filtered. The resulting clean reads were aligned to the rice reference genome IRGSP-1.0 (https://rapdb.dna.affrc.go.jp) using HISAT2 version 2.09 [19], and the number of reads mapped to each gene was counted with featureCounts (v1.5.0-p3) [20]. The expression level of each gene was calculated as fragments per kilobase of transcript sequence per million base pairs sequenced (FPKM) values. Differential gene expression analysis between samples was performed using DESeq2 R package v. 1.18.0 [21]. Genes with an absolute value of log_2_(fold change) > 0 and a false discovery rate (FDR) adjusted *p* value (*padj*) < 0.05 using the Benjamini–Hochberg method were defined as differentially expressed genes (DEGs). The iTAK (plant transcription factor and protein kinase identifier and classifier) program [22] was used to identify transcription factors (TFs) and transcriptional regulators (TRs). Gene ontology (GO) and the Kyoto Encyclopedia of Genes and Genomes (KEGG) enrichment analyses of the DEGs were performed using the clusterProfiler R package [23] with *padj* < 0.05 to find significant enrichments.

### 2.5. Metabolite Profiling

Metabolome analyses of the leaf samples were performed as previously described [24]. Briefly, the freeze-dried leaf samples were grinded using a mixer mill (MM 400, Retsch, Haan, Germany) for 1.5 min at 30 Hz. For each sample, 100 mg of powder was measured and incubated overnight at 4 °C with 1 mL of 70% aqueous methanol. After centrifugation at 10,000× *g* for 10 min, the extracts were absorbed (CNWBOND Carbon-GCB SPE Cartridge, 250 mg, 3 mL; ANPEL, Shanghai, China), filtered (SCAA-104, 0.22 μm pore size; ANPEL, Shanghai, China), and stored in a glass vial for the following analysis. LC-MS analysis was conducted using a liquid chromatography-electrospray ionization-tandem mass spectrometry (LC-ESI-MS/MS) system (high-performance liquid chromatography (HPLC): Shim-pack UFLC Shimadzu CBM20A system; MS, Applied Biosystems 4000 Q TRAP), as previously described [25]. Identification of metabolites was performed using the MetWare Database (Wuhan MetWare Biotechnology Co., Ltd. Wuhan, China) and publicly available metabolite databases, including MassBank (http://www.massbank.jp/), KNAPSAcK (http://kanaya.naist.jp/KNApSAcK/), HMDB (http://www.hmdb.ca/), MoTo DB (http://www.ab.wur.nl/moto/), and METLIN (http://metlin.scripps.edu/index.php). Metabolite quantification was performed using a scheduled multiple reaction monitoring method in triple quadrupole mass spectrometry [25].

### 2.6. Metabolite Data Analysis

The supervised multivariate method, orthogonal projections to latent structures discriminant analysis (OPLS-DA), was used to determine differentially accumulated metabolites (DAMs) between leaves fed upon by *C. medinalis* and control leaves. The relative importance of each metabolite to the OPLS-DA model was checked using the variable importance in projection (VIP) metric [24]. Metabolites with VIP ≥ 1.0 and fold change ≥ 2 or fold change ≤ 0.5 were considered as DAMs for group comparations.

### 2.7. Phytohormone Analyses

#### 2.7.1. Phytohormone Signatures Analyses

To identify the transcriptional signatures of hormonal responses of rice against *C. medinalis*, the Hormonometer program [26] was used to compare the similarity of expression of rice genes with those elicited by exogenous application of phytohormones to *Arabidopsis thaliana*. As the comparation was conducted with *Arabidopsis*, we selected orthologous genes between the *Arabidopsis* and rice genomes. Only orthologous genes detected in the RNA-seq that related to *Arabidopsis* probe set identifiers were selected for further analyses.

#### 2.7.2. Determination of Endogenous JA and JA-Isoleucine Levels

The endogenous stress-related hormone JA and the biologically active metabolite JA-Ile were extracted at different time points (0, 1, 6, 12, and 24 h) from rice leaves and detected by MetWare (MetWare Biotechnology Co., Ltd., Wuhan, China). Briefly, frozen rice leaves were ground into fine powder using a mixer mill (MM 400, Retsch, Haan, Germany) for 1.5 min at 30 Hz. Then, 50 mg of the powdered leaf samples were extracted with methanol/water/formic acid (15:4:1, V/V/V) at 4 °C overnight, and ultrasound-assisted extraction was conducted at room temperature for 30 min with vortexing (15 s), followed by centrifugation at 14,000 rpm for 10 min. A total of 1000 μL of supernatant was collected, evaporated to dryness with nitrogen gas at room temperature, reconstituted in 100 μL of 80% (*v/v*) methanol, and diluted to 800 μL with water. Extracts were then passed through a SPE cartridge (CNWBOND carbon-GCB, 250 mg, 3 mL; ANPEL, Shanghai, China) and evaporated to dryness using nitrogen gas at room temperature. The samples were reconstituted in 200 μL of 80% (*v/v*) methanol, filtered (SCAA-104, 0.22 μm pore size; ANPEL, Shanghai, China), and stored in glass vials before analysis. The samples were analyzed using a liquid chromatography-electrospray ionization-tandem mass spectrometry (LC-ESI-MS/MS) system (high-performance liquid chromatography (HPLC), Shim-pack UFLC Shimadzu CBM20A system, Kyoto, Japan; MS, Applied Biosystems 4000 Q TRAP, Foster City, CA, USA), as previously described [27]. Three replicates for each time point were analyzed.

### 2.8. Statistical Analysis

Analysis of the patter of expression of all DEGs was conducted using Multi Experiment Viewer (MeV version 4.9.0 (http://mev.tm4.org/#/welcome)) by a *K*-means algorithm based on the log_2_ values of FPKM. Pearson’s correlation was used to compute similarity distance in MeV software. Gene expression data and metabolite accumulation data were normalized using MetaboAnalyst 4.0 [28], with the following parameters: normalization by median, log transformation, and auto scaling (mean-centered and divided by the standard deviation of each variable). A hierarchical clustering heatmap was generated using MetaboAnalyst 4.0 with the Euclidean distance measure and Ward clustering algorithm. The normalized transcriptome and metabolome datasets were imported to SIMCA 14.1 software package (Umetrics, Umea, Sweden), and principal component analysis (PCA) was conducted to acquire an overview of the data. Venn diagrams were generated using the Venny 2.1.0 drawing tool (http://bioinfogp.cnb.csic.es/tools/venny/index.html). Student’s *t*-test was used to compare the contents of JA and JA-Ile in rice leaves between different time points and the control (0 h) using SPSS 22.0 software (IBM SPSS, Somers, NY, USA), and a significance level of *p* < 0.05 was applied.

### 2.9. Data Availability

The transcriptome sequencing raw data used in this study was submitted to the NCBI’s Gene Expression Omnibus (GEO) under accession number GSE159259. The metabolome data can be found within the manuscript and the supporting materials.

## 3. Results

### 3.1. Overview of Transcriptome and Metabolome Dataset

During the 24 h of feeding, *C. medinalis* caterpillars caused clear damage to rice leaves (Figure S1). Global transcriptome changes in rice leaves, responding to 1, 6, 12, and 24 h of such feeding, were quantified by examining mRNAs of 20 libraries (four biological replicates at each of the five sampling times) that collectively included 164.04 Gb of clean reads. Each of these samples contained at least 7.36 Gb of data with Q30 quality scores ≥ 90.70% (Table S2). The GC content accounted for 53.16–53.71% of these reads. A total of 44.84–56.19 million reads were mapped uniquely to the rice IRGSP-1.0 reference genome, with unique mapping rates ranging from 89.73% to 91.36% (Table S2). Gene structure analyses showed that most of the mapped reads (92.40–95.70%) were distributed in exons (Table S2). The unique mapped reads were used for the following analyses.

The leaf transcriptome data was validated by qRT-PCR analyses using samples from the same batch of rice plants that were used for RNA-seq. The results showed that the expression of the selected eight JA pathway-associated genes were consistent with those analyzed by RNA-seq (Figure S2), which indicated that the transcriptome datasets were sufficient for further analyses.

**Figure 2:**
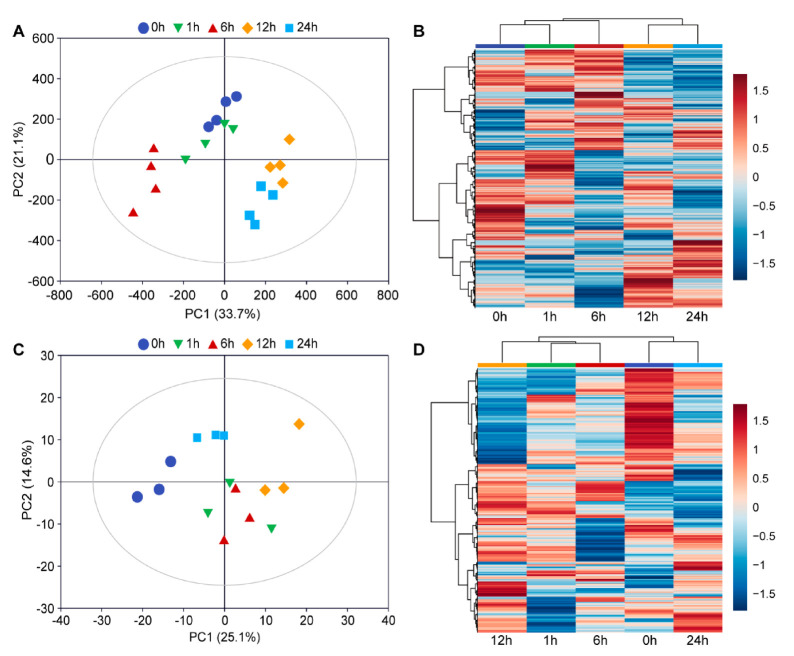
Overview of rice transcriptome and metabolome changes upon feeding by *C. medinalis*. (**A**,**C**) Principal component analysis (PCA) plot of all detected transcripts and metabolites in MH63 rice leaves by RNA-sequencing and metabolome profiling. Data points indicate the respective time points as feeding progressed. (**B**,**D**) Cluster analysis and heat maps of relative levels of expression of all transcripts and metabolites detected at different time points. Color coding represents the intensity of induction (red) or suppression (blue) of gene expression by insect feeding.

RNA-seq data were normalized to FPKM values to quantify gene expression levels. PCA of all detected genes showed that the first two principal components (PCs) explained 54.8% of the total variation (Figure 2A). In particular, biological replicates from the same time point were clustered together, which suggested good repeatability for each treatment. The samples from different time points were clustered far away from each other, which indicated that the time that the leaves had been subjected to *C. medinalis* infestation posed different effects on rice transcriptome profiles. Samples from the 1-h treatment clustered near those of the 0 h (control) samples, and samples from the 12-h treatment clustered near those of the 24-h treatment. Samples from the 6-h treatment clustered further from the 1-, 12-, and 24-h time points, indicating that rapid change in gene expression occurred after the onset of *C. medinalis* feeding. The hierarchical clustering heatmap (Figure 2B) showed that samples from *C. medinalis* feeding at early stages (1 and 6 h) and late stages (12 and 24 h) were clustered together, respectively.

Metabolomic changes in *C. medinalis*-infested rice leaves were investigated using LC-ESI-MS/MS. A total of 502 metabolites were detected among samples collected from the four time points. These metabolites were grouped into 11 classes, with the majority belonging to the classes of flavonoids (179), phenolic acids (61), and amino acids and derivatives (52) (Table S3). In addition, a few metabolites from the classes of terpenoids (2) and tannins (1) were also detected. Results of PCA clustering of the metabolites detected at different time points are presented in Figure 2C. PCA results showed that the first two PCs explained 39.7% of the total variation. The plot also showed distinct separation of metabolomes of rice plants infested by *C. medinalis* from those of the controls. The hierarchical clustering heatmap (Figure 2D) indicated that samples at 1, 6, and 12 h were cluster together and were separated from samples at 24 h and the control plants. In contrast to gene expression, metabolomic samples from 0 and 24 h were clustered together.

### 3.2. Analyses of Differentially Expressed Genes and Differentially Accumulated Metabolites

**Figure 3:**
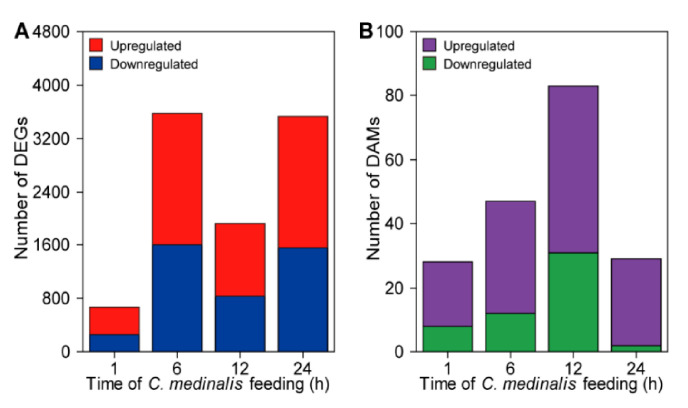
Numbers of (**A**) differentially expressed genes (DEGs) and (**B**) differentially accumulated metabolites (DAMs) that are up- and downregulated in rice leaves damaged by *C. medinalis* at different time points compared to undamaged control plants.

**Figure 4:**
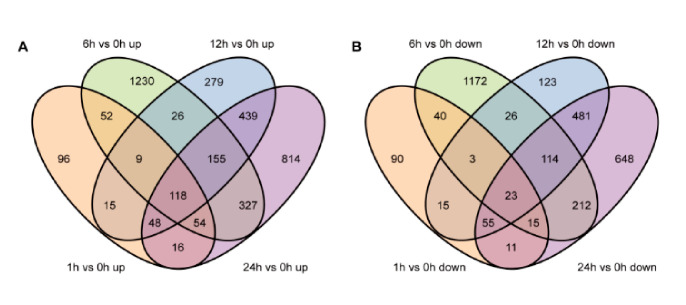
Venn diagrams illustrating the unique and shared differentially expressed genes that were (**A**) up- or (**B**) downregulated by *C. medinalis* infestation through the time course.

Using the absolute value of log_2_(fold change) > 0 and the FDR adjusted *p* value (*padj*) ≤ 0.05 as thresholds for recognizing DEGs between any two treatments, a total of 6372 DEGs were identified in rice leaves at different time points (1, 6, 12, and 24 h) of *C. medinalis* infestation (Tables S3 and S4). After 1, 6, 12, and 24 h of feeding, respectively, 660 (408 up- and 252 downregulated), 3575 (1971 up- and 1605 downregulated), 1929 (1089 up- and 840 downregulated), and 3530 (1971 up- and 1559 downregulated) DEGs were identified (Figure 3A, Table S4). The distribution of these up- and downregulated genes was calculated and presented in a Venn diagram (Figure 4). A unique set of genes were up- (1230) and downregulated at 6 h (1172). In addition, a total of 3608 genes showed increased expression, of which 118 genes were significantly upregulated at all time points (Figure 4A), while there were 3028 genes showing decreased expression, of which only 23 genes were downregulated at all four time points (Figure 4B).

Metabolite abundance increased more slowly than gene expression (Figure 3B). The highest number of DAMs was detected at 12 h after initiation of *C. medinalis* feeding. More metabolites were upregulated than downregulated in response to *C. medinalis* infestation. The distribution of these DAMs in different time points was compared and presented using a Venn diagram (Figure S3a). Specifically, only three compounds were significantly affected by *C. medinalis* feeding at all four time points, and their accumulation is shown in Figure S3b.

### 3.3. Identification of Calcium Ions Sensors, Transcription Factors, and Transcriptional Regulators That Responded to C. medinalis Feeding

Early signaling events, such as calcium ion (Ca^2+^) sensors and proteins that regulate the expression of target genes, such as transcription factors (TFs) and transcriptional regulators (TRs), play important roles in plant responses to insect herbivores [1,2,6]. Because these signals activate downstream pathways in response to insect-mediated stress, observation of their induction can provide insight into molecular mechanisms of plant response to insect herbivory stress. Among the DEGs, we identified three calmodulins (CaMs), 14 calmodulin-like proteins (CMLs), and 22 calcium-dependent protein kinases (CDPKs) (Figure S4). In addition, 612 TFs or TRs were identified among rice genes whose expression was detected. Among these, 497 TFs or TRs distributed among 64 families were significantly differentially expressed in at least one time point (Table S4, Figure S5). These TFs or TRs mainly included the following families: 40 myeloblastosis (MYB) genes, 35 apetala2-ethylene-responsive element binding protein (AP2-EREBP) genes, 32 basic helix-loop-helix (bHLH) genes, 28 NAM, ATAF1-2, and CUC2 (NAC) genes, and 24 WRKY genes (Table S4, Figures S5 and S6).

### 3.4. GO and KEGG Enrichment Analysis of DEGs Induced by C. medinalis Feeding

**Figure 5:**
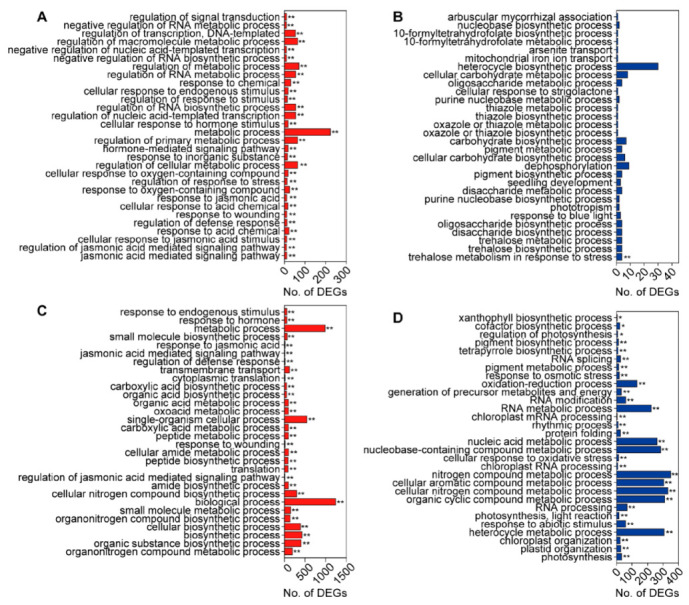
The top 30 enriched gene ontology (GO) terms of the differentially expressed genes (DEGs) in rice leaves infested with *C. medinalis* at early post-infestation (1 h) and late post-infestation (24 h) relative to control plants. (**A**,**C**) GO enrichment analysis of upregulated DEGs at 1 and 24 h post-infestation, respectively. (**B**,**D**) GO enrichment analysis of downregulated DEGs at 1 and 24 h post-infestation, respectively. Asterisks indicate statistically significant differences: * *padj* < 0.05, ** *pddj* < 0.01 via the Benjamini and Hochberg method.

GO enrichment analysis was used for functional classification of the DEGs in rice leaves, responding to feeding by *C. medinalis*. Significantly enriched biologically processed GO terms for genes that were differentially expressed at the early time point (1 h) and late time point (24 h) are shown in Figure 5. At 1 h, upregulated genes were involved in the “jasmonic acid mediated signaling pathway”, “regulation of jasmonic acid mediated signaling pathway”, “cellular response to jasmonic acid stimulus”, “response to acid chemical”, and “regulation of defense response”; the downregulated DEGs were only involved in “trehalose metabolism in response to stress” (Figure 5A,B). At 24 h, upregulated DEGs were significantly enriched in 76 GO biological terms, which included the “organonitrogen compound metabolic process”, “organic substance biosynthetic process”, “biosynthetic process”, “cellular biosynthetic process”, and “organonitrogen compound biosynthetic process”. In particular, the upregulated DEGs also were enriched in GO terms associated with JA pathways (Figure 5C). The downregulated DEGs at 24 h were significantly enriched in “photosynthesis”, “plastid organization”, “chloroplast organization”, “heterocycle metabolic process”, and “response to abiotic stimulus” (Figure 5D). The full list of significantly enriched GO terms for DEGs at different time points is shown in Table S6.

KEGG enrichment analyses showed that both the upregulated and downregulated genes at 1 h were significantly enriched in plant hormone signal transduction. The upregulated genes at 6 h were significantly enriched in “alpha-linolenic acid metabolism” and amino acid metabolism, and the downregulated genes were significantly enriched in terms associated with photosynthesis. At 12 and 24 h, the upregulated genes were enriched in defense-associated terms, such as “biosynthesis of secondary metabolites”, “diterpenoid biosynthesis”, and “biosynthesis of amino acids”, while photosynthesis-related terms, including “photosynthesis-antenna proteins” and “porphyrin and chlorophyll metabolism”, were enriched among the downregulated genes (Table S6).

### 3.5. Clustering Analyses of the Transcriptome Dataset

**Figure 6:**
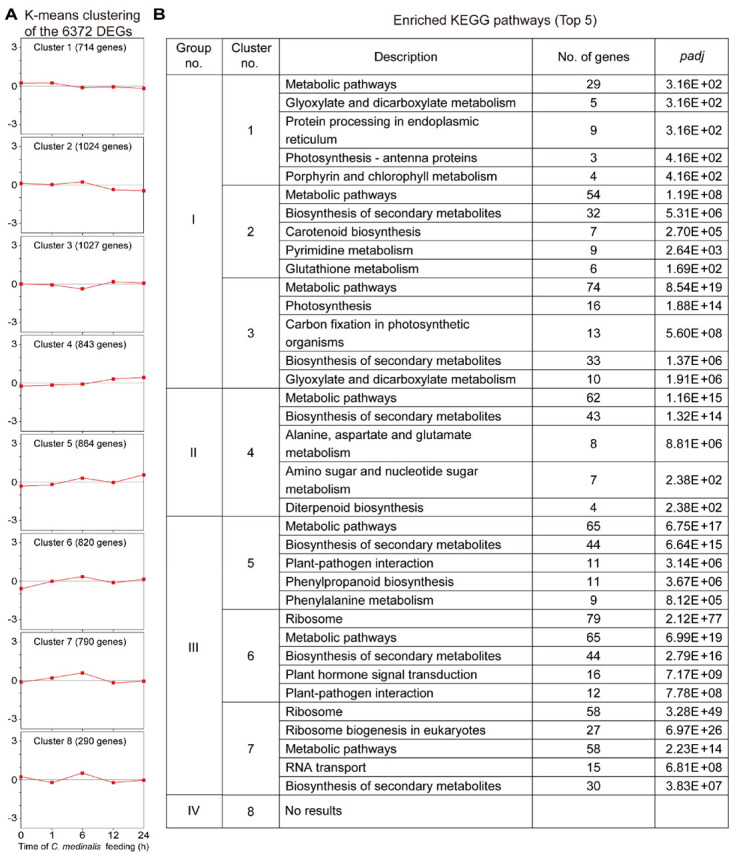
Kyoto Encyclopedia of Genes and Genomes (KEGG) enrichment analysis of differentially expressed genes (DEGs) grouped by the *K*-means clustering method. (**A**) *K*-means clustering of differentially expressed genes scaled as Z-score. Eight clusters involving 6372 DEGs induced by *C. medinalis* feeding. The number of genes in each cluster is indicated. The expression data for individual genes are shown in light grey, and the average expression for each cluster is shown in red. The x-axis shows the different feeding stages and the y-axis shows the Z-scores. (**B**) KEGG enrichment analysis of genes in each cluster. The top five significantly enriched pathways are listed for each cluster. “No results” indicates that there were no significantly enriched KEGG terms.

To discover the molecular mechanisms of rice response to *C. medinalis* feeding, DEGs at the four time points were clustered using the *K*-means method. Eight clusters were obtained, and each cluster was represented by the Z-score of gene expression from the sets of genes with similar expression patterns (Figure 6). The eight clusters were divided into four groups according to the gene expression trends in each cluster: Group I) clusters 1, 2, and 3 include genes with reduced levels of expression through the times of infestation. Cluster 2 presents genes with minor upregulation at 6 h, and cluster 3 represents genes that were upregulated at 12 h; Group II) cluster 4 includes genes with increased levels of expression throughout the feeding times; Group III) clusters 5, 6, and 7 include genes with increased levels of expression at early time points but reduced levels at late time points. Clusters 5 and 6 represent genes that were downregulated at 12 h, and cluster 7 represents genes with reduced levels at 12 and 24 h; Group IV) cluster 8 represents genes that were downregulated at 1 h, with increased levels at 6 h and reduced levels at 12 h. The distribution of genes associated with the eight clusters is presented in Table S8.

To elucidate the metabolic processes within each cluster, we conducted KEGG pathway enrichment analysis (Figure 6, Table S9). The results showed that genes in Group I were significantly enriched in pathways associated with “metabolic pathways”, “biosynthesis of secondary metabolites”, and “photosynthesis”. Genes in Group II were significantly enriched in defense-related pathways, such as “biosynthesis of secondary metabolites” and “alanine, aspartate, and glutamate metabolism”. Genes in Group III were significantly enriched in pathways associated with plant secondary metabolism, “plant hormone signal transduction”, and “plant-pathogen interaction”. There were no particular pathways that were significantly enriched in the genes belonging to Group IV. Taken together, these results indicate that the *C. medinalis* feeding activated complex changes in rice plant metabolism associated with growth and defense.

### 3.6. Plant Hormone-Related Genes Induced by C. medinalis Feeding

**Figure 7:**
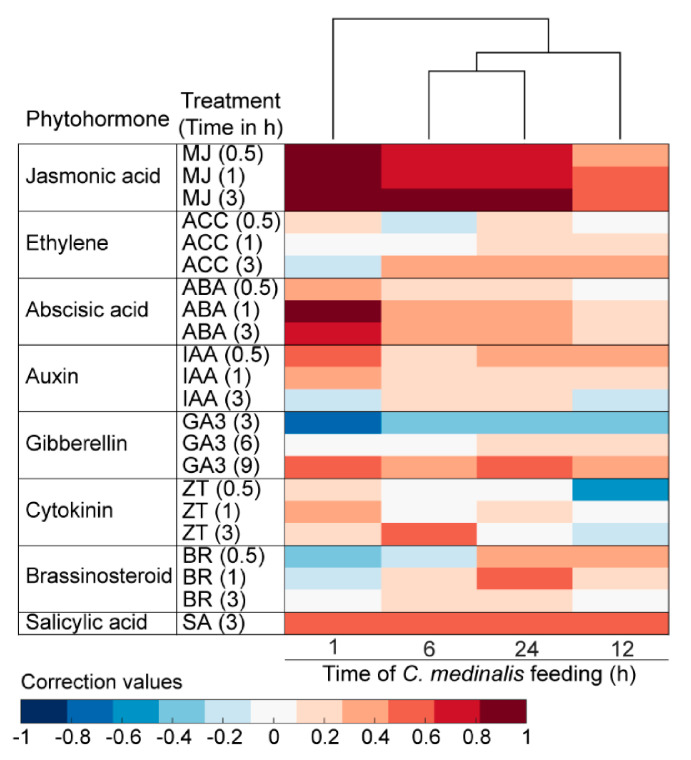
Comparison of plant hormone signatures between transcriptomic data generated after onset of *C. medinalis* feeding on rice leaves and *Arabidopsis* response to hormone treatments. Red shading indicates positive and blue shading indicates negative correlations between the rice response to feeding by *C. medinalis* and the *Arabidopsis* response to the respective hormone treatments: methyl jasmonate (MJ); 1-aminocyclopropane-1-caroxylic acid (ACC; precursor of ethylene); abscisic acid (ABA); indole-3-acetic acid (IAA); gibberellic acid 3 (GA3); zeatin ZT); brassinosteroid (BR); and salicylic acid (SA).

GO, KEGG enrichment, and clustering analyses indicated that rice phytohormone signaling pathways were differentially affected by feeding of *C. medinalis*. We therefore evaluated similarities in the expression of genes responding to *C. medinalis* infestation and those induced by application of plant hormones in *Arabidopsis*. A total of 15,996 *Arabidopsis* orthologs of rice genes were used for the Hormonometer analysis (Table S10). The results showed that expression of genes associated with JA- and ABA-dependent signaling was highly induced after 1 h of infestation, with more moderate induction of these phytohormone signaling pathways at subsequent time points (Figure 7). Expression of genes associated with brassinosteroid and cytokinin at the early (1 h) and middle (12 h) infestation stages showed negative correlations with those responses to the application of plant hormones in *Arabidopsis*. Interestingly, expression of gibberellin-responsive genes was negatively correlated with genes whose expression in *Arabidopsis* was elicited 3 h after gibberellin treatment. These results suggested that genes associated with the JA pathway were most induced by *C. medinalis* infestation.

**Figure 8:**
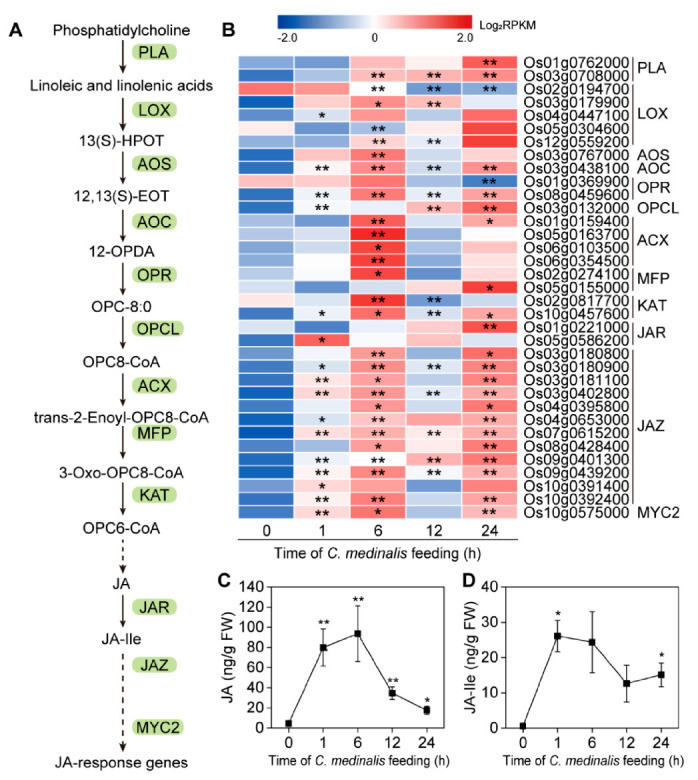
*C. medinalis*-induced responses in the jasmonic acid (JA) pathway. (**A**) Overview of the JA pathway. (**B**) Heat map of the expression of genes associated with the JA pathway. Asterisks indicate a significant difference of the expression of genes at different time points relative to control (0 h): * *padj* < 0.05, ** *padj* < 0.01 via the Benjamini and Hochberg adjustment method. (**C**) JA and (**D**) JA-isoleucine conjugate (JA-Ile) contents (ng/g FW) in rice leaves. Values are mean ± SE of three biological replicates. Asterisks indicate statistically significant differences compared to time zero: * *p* values < 0.05, ** *p*-values < 0.01 (Student’s *t* test). phospholipase A1 (PLA); lipoxygenase (LOX); 13(S)-hydroperoxylinolenic acid (13(S)-HPOT); allene oxide synthase (AOS); 12,13(S)-epoxylinolenic acid 12,13(S)-EOT); allene oxide cyclase (AOC); 12-oxo-phytodienoic acid (12-OPDA); 12-oxophytodienoate reductase (OPR); 3-oxo-2-(2′(Z)-pentenyl)-cyclopentane-1-octanoic acid (OPC-8:0); OPC8-CoA ligase (OPCL); acyl-CoA oxidase (ACX); multifunctional protein (MFP); 3-ketoacyl-CoA thiolase (KAT); jasmonate resistant (JAR); jasmonate-ZIM domain (JAZ).

To determine the role of the JA pathway in response to *C. medinalis* feeding, we analyzed the expression of the genes involved in the JA pathway and detected the concentrations of JA and JA-Ile in rice leaves at 0 h, 1 h, 6 h, 12 h, and 24 h following infestation (Figure 8). As shown in Figure 8B, most of the genes involved in the biosynthesis and signaling of JA and JA-Ile were significantly induced by *C. medinalis* feeding, with some key genes, such as *AOC* (*Os03g0438100*), which were induced 1 h after *C. medinalis* infestation. Accordingly, the levels of JA and JA-Ile in affected plants increased significantly compared to control plants, and both JA and JA-Ile reached a peak value within a short time (Figure 8C,D).

## 4. Discussion

The development of omics-based high-throughput experimental approaches, such as genomics, transcriptomics, proteomics, and metabolomics profiling, has provided enhanced ability to characterize plant-defense responses to abiotic and biotic factors, including insect attack [5]. For example, transcriptomics makes it possible to obtain transcriptional maps at the whole-genome levels and metabolomics has been used to qualify and quantify low molecular weight metabolites in the cells of an organism. Due to the highly complex hierarchical organization of plant systems, transcriptomics has the potential to quantify changes in gene expression and associated regulatory mechanisms, while metabolomics supports the study of the biochemical characteristics of metabolites. In recent years, the integration of transcriptomics and metabolomics analyses has been applied to many agricultural plant species, such as maize, rice, cotton, and wheat, for identification of the gene-to-metabolites network associated with insect defense [17,29,30,31,32]. In the current study, an integrated transcriptomic and metabolomic approach was applied to characterize the defense response of rice leaves to *C. medinalis* infestation.

Generally, plant defense against insect herbivore attack is a multi-dimensional dynamic process and involves many levels of organizational and functional complexity, executed at different time scales, ranging from fractions of seconds to millions of years [5,6]. For example, in maize leaves, feeding by the beet armyworm (*Spodoptera exigua*) caterpillar and corn leaf aphid (*Rhopalosiphum maidis*) induced 2449 DEGs and more than 900 DEGs, respectively, within 2 h [29,33]. Consistent with the results of previous studies, we found that *C. medinalis* feeding caused obvious damage on rice leaves in a short time (<6 h) and induced a rapid defense response. At the early stage (1 h), a total of 660 genes and 28 DAMs were significantly up- or downregulated by caterpillar infestation. After 24 h of feeding, there were more than 3500 genes and 29 DAMs were significantly up- or downregulated. Interestingly, although similar numbers of DEGs were detected at 6 and 24 h, there was only a small overlap of the DEGs between these time points. These results suggest that a tightly controlled defense response is executed in rice plants, which regulates the expression of various genes at different time points to launch an optimal defense process. In particular, after 24 h of *C. medinalis* caterpillar feeding, the metabolomic pattern in the rice leaves is closer to that in control plants compared with plants infected by caterpillars for 1, 6, or 12 h (Figure 2D), which may suggest that metabolic responses in plants can be induced rapidly upon insect infestation, then trend to alleviation with prolonging infestation time. Similar, time point-specific variation in plant gene expression and metabolite accumulation has been observed in other plant–insect interactions [17,29,33]. The findings obtained in the current study, together with previous studies, indicate dynamic shifts in plant defense against insect feeding over time and suggest that rice and other plants are able to fine-tune their responses at both mRNA and metabolite levels over time to defend insect herbivore attack.

As a plant becomes subject to insect herbivory, early signaling events play a vital role in the induction of herbivore-associated molecular responses. The transient rise in the concentration of cytosolic calcium ion (Ca^2+^) is one of the main signals used by plants in perception of insect herbivores. The transmission of Ca^2+^ signals is mediated by Ca^2+^ sensors, including CaMs, CMLs, and CDPKs [6]. In the current study, we detected a rapid response in expression of *CaMs* (*Os03g0319300*) and CDPK (*Os12g0113500*, *Os08g0540400*, and *Os08g0441100*) genes in rice leaves as early as 1 h after the onset of *C. medinalis* feeding. Moreover, most CML genes were activated at late feeding stages. In *Arabidopsis*, feeding by green peach aphid (*Myzus persicae*) and cotton leafworm (*Spodoptera littoralis*) elicits a rapid, significantly elevated concentration of calcium ions around the feeding sites within 95 s after infestation [34,35]. Several CML genes, including *CML9*, *CML11*, *CML12*, *CML16*, *CML17,* and *CML23*, are upregulated by the application of oral secretion from *S. littoralis* [36]. *CML37* positively regulates *S. littoralis-*induced defense responses in *Arabidopsis* by activating the JA pathway [37], while CML42 negatively regulates plant defense by suppressing the JA pathway and aliphatic glucosinolate contents [38]. Interestingly, although *CML9* is significantly induced by *S. littoralis* infestation, it is not involved in *Arabidopsis* defense against this pest and contributes to the defense against biotrophic pathogens independent of the JA pathway [39]. Currently, the functions of Ca^2+^ sensors in plant-herbivore interactions has mainly been investigated in *Arabidopsis*, and hence the role of Ca^2+^ sensors in rice defense against herbivores requires further investigation.

JA plays central roles in regulating plant defense against insect herbivore attack [2,6,7] and was also indicated in the current study. Our results showed that feeding, by *C. medinalis* at all four time points, activated genes were involved in the KEGG pathway “alpha-Linolenic acid metabolism”. As expected, accumulation in the contents of both JA and JA-Ile was detected at these time points. It has been shown that silencing of genes involved in the biosynthesis of JA in rice, such as *OsHI-LOX* and *OsPLDa4/5*, results in decreased JA levels and trypsin protein inhibitor (TrypPI) content and thus promotes the growth and development of rice striped stem borer *Chilo suppressalis* [40]. Furthermore, silencing of genes such as *OsCOI1* in rice plants decreases TrypPI contents and the enzymatic activities of polyphenol oxidase and peroxidase and increases susceptibility to *C. medinalis* [41]. In turn, application of MeJA and JA on rice plants activates diverse defense metabolites, including polyphenol oxidase, TrypPI, and plant volatiles, thereby leading to the direct and indirect resistance of plants to herbivores [14,42,43]. Previously, it has been shown that *C. medinalis* infestation suppresses the endogenous SA in rice plants [18]. Our results, together with those of previous reports, suggested that an antagonistic interaction of JA and SA might be involved in rice defense against *C. medinalis*. However, synergistic JA-SA crosstalk is also reported in rice plants [7,17]. For example, in the OsHPL3-mediated oxylipin pathway, synergistic activation of the JA and SA signaling pathways confers resistance against bacterial blight (*Xanthomonas oryzae* pv*. oryzae*) in rice. Moreover, it was also suggested that there were overlapping or even synergistic effects between SA and JA in rice defense against *C. suppressalis* [17]. Taken together, JA and its crosstalk with other hormones in rice is crucial for plant-specific defense against herbivory.

A number of studies have shown that the mitogen activated protein kinase (MAPK) signaling pathway plays critical roles in plants’ resistance to herbivores [1,44]. Transcription factors (TFs) also play important roles in plant responses to herbivory stress, and, specifically, the WRKY TFs are involved in the MAPK pathways in rice plants [31,44]. In this study, the expression of several MAPKs, including *OsMPK3* (*Os03g0285800*) and mitogen-activated protein kinase kinases (MAPKKs), were significantly upregulated by *C. medinalis* feeding, indicating that MAPK signaling plays critical roles in regulating the induced defense responses of rice to this pest. In addition, the expression levels of many WRKY genes, such as *OsWRKY53* (*Os05g0343400*), *OsWRKY24* (*Os01g0826400*), and *OsWRKY45* (*Os05g0322900*), were significantly upregulated by *C. medinalis* infestation. After the onset of insect feeding, the WRKYs were phosphorylated by different MAPKs, and they, in turn, regulated expression of downstream stress-response genes [44]. In rice, OsWRKY70 physically interacts with and is regulated by OsMPK3 and OsMPK6 and prioritizes defense over growth by positively regulating JA and negatively regulating gibberellin biosynthesis upon attack by *C. suppressalis* [45]. OsWRKY53 physically interacts with OsMPK3 and OsMPK6 and suppresses their activity, which results in decreased levels of *C. suppressalis*-induced JA, JA-Ile, ethylene, and TrypPI, thereby negatively regulating rice defense against *C. suppressalis* [46]. Moreover, OsWRKY45 was found to negatively modulate the resistance of rice to brown planthopper *Nilaparvata lugens* [47]. Although several studies have verified the roles of MAPK-WRKY pathways in rice defense responses against insects, such as *C. suppressalis* and *N. lugens*, their roles in rice defense against *C. medinalis* are still unclear. Moreover, the underlying molecular mechanisms and roles of different WRKYs in induced defense processes in rice need further investigation.

As a leaf-feeding insect, *C. medinalis* larvae stay inside the folded leaf and feed by scraping the green mesophyll tissue, resulting in linear membranous damage (Figure S1) and reduced photosynthetic efficiency [12]. Our transcriptomic analysis provided molecular evidence that the downregulated genes induced by *C. medinalis* feeding were significantly enriched in photosynthesis-related GO terms, such as “photosynthesis”, “photosynthesis, light reaction”, “photosynthesis, light harvesting”, and “response to light stimulus”, and also in KEGG terms, including “photosynthesis” and “photosynthesis-antenna proteins”. Similar results also were reported in wheat, in which genes involved in photosynthesis, such as asribulose-1,5-bisphosphate carboxylase and chlorophyll A-B binding protein, were significantly downregulated by feeding of the greenbug *Schizaphis graminum* [48]. In plants, the downregulation of the photosynthetic apparatus could protect them from oxidative damage [49]. In addition, production of the photosynthetic apparatus is energy-intensive, and, as a result of a trade-off for the synthesis of defense metabolites, photosynthesis was generally compromised [50]. Moreover, the decrease of photosynthetic activity also may free up resources, especially nitrogen-rich compounds, making them available for use in secondary defense pathways [17,49,50].

## 5. Conclusions

In summary, we applied RNA-seq and metabolome profiling in this study, revealing dynamic transcriptional and metabolite changes in rice leaves in response to a *C. medinalis* caterpillar attack. Feeding by this herbivore induced rapid and precise defense responses involved in many primary and secondary metabolic processes in rice leaves. Our results suggest that the JA-dependent signaling pathway triggered by feeding of *C. medinalis* plays vital roles in the response of rice plants. Future studies should focus on the roles and underlying molecular mechanisms of early signaling events and TFs in this process. This study extends the understanding of the molecular mechanisms involved in rice defense against rice leaf folder and may facilitate the development of insect-resistant rice varieties by identifying molecular targets for selection.

## Supplementary Materials

The following are available online at https://www.mdpi.com/2075-4450/11/10/705/s1, Figure S1: Phenotypes of rice leaves infested by *C. medinalis* at different time points. Figure S2: Comparison of mRNA expression levels detected by RNA-seq and qRT-PCR. All qRT-PCR data were normalized against that of the housekeeping gene *ubiquitin 5*. Values are means ± SE; *n* = 4 for RNA-seq and *n* = 3 for qRT-PCR. Specific information on these genes is provided in Table S1. Figure S3: The distribution of differentially accumulated metabolites (DAMs) at different time points. (a) Venn diagrams illustrating the unique and shared DAMs among the four time points. (b) Heatmap of the accumulation of three common compounds induced by *C. medinalis* feeding. Index of compounds, mws0064: eriodictyol, mws0885: 2,4-dihydroxy benzoic acid, pme3475: butin. Figure S4: Heat map of the expression of calcium ion sensor genes induced by *C. medinalis* feeding at different time points. Asterisks indicate a significant difference in the expression of genes at different time points relative to control (0 h): * *padj* < 0.05, ** *padj* < 0.01 via the Benjamini and Hochberg adjustment method. Figure S5: Distribution of transcription factors (TFs) and transcriptional regulator (TR) families identified in differentially expressed genes (DEGs). Figure S6: Heat map of the expression of DEGs involved in mitogen-activated protein kinase (MAPK)-WRKY pathways in response to *C. medinalis* feeding at different time points. Asterisks indicate a significant difference in the expression of genes in different time points relative to control (0 h): * *padj* < 0.05, ** *padj* < 0.01 via the Benjamini and Hochberg adjustment method. Table S1: Genes and primer pairs used for quantitative real-time PCR. Table S2: Summary for the transcriptome dataset. Table S3: Metabolic profiles for compounds accumulated in rice leaves collected from different time points. Table S4: DEGs in rice leaves in response to *C. medinalis* feeding at different time points. Table S5: Differentially expressed transcription factors and transcriptional regulators in rice leaves in response to *C. medinalis* feeding. Table S6: GO enrichment analysis of the total DEGs of rice leaves in response to *C. medinalis* feeding at different time points. Table S7: KEGG enrichment analysis of the upregulated and downregulated DEGs in rice leaves in response to *C. medinalis* feeding at different time points. Table S8: Clustering analyses of DEGs in rice leaves in response to *C. medinalis* feeding at least one time point. Table S9: KEGG analyses of DEGs belong to different clusters. Table S10: Orthologous *Arabidopsis* and rice genes used for Hormonometer analysis.
